# Supplementary material for: Polyketide synthase gene clusters in the ericoid mycorrhizal fungus Oidiodendron maius: genome-wide prediction, expression profiling, and functional characterization of knockout mutants
Source: Front Plant Sci. 2026 Sep 16;17:1924430. doi: 10.3389/fpls.2026.1924430 (PMC13623903; doi:10.3389/fpls.2026.1924430)
Supplement: Supplementary file 1 [file SupplementaryFile1.zip › Supplementary Materials/Supplementary Folder 1.zip!/Oidma1_AssemblyScaffolds_Repeatmasked/knownclusterblast/cluster2/jgi.p_Oidma1_139660_mibig_hits.html]

| MIBiG Protein | Description | MIBiG Cluster | MiBiG Product | % ID | % Coverage | BLAST Score | E-value |
| --- | --- | --- | --- | --- | --- | --- | --- |
| ACD39772.1 | major\_facilitator\_superfamily\_transporter | BGC0000134 | Radicicol\_biosynthetic\_gene\_cluster | 31 | 90.9584086799 | 212 | 1e-54 |
| ADI24938.1 | VrtL | BGC0000168 | Viridicatumtoxin\_biosynthetic\_gene\_cluster | 32 | 69.6202531646 | 207 | 5.7e-53 |
| BAK26557.1 | putative\_MFS\_multidrug\_transporter | BGC0000977 | Cyclopiazonic\_acid\_biosynthetic\_gene\_cluster | 31 | 66.7269439421 | 165 | 1.5e-40 |
| EWG54280.1 | hypothetical\_protein | BGC0001190 | Fusaric\_acid\_biosynthetic\_gene\_cluster | 41 | 24.0506329114 | 108 | 2.8e-23 |
| KFH48645.1 | putative\_transporter\_-like\_protein | BGC0000317 | Cephalosporin\_biosynthetic\_gene\_cluster | 41 | 23.6889692586 | 107 | 6.1e-23 |
| CAP95407.1 |  | BGC0001404 | Sorbicillin\_biosynthetic\_gene\_cluster | 39 | 23.869801085 | 93 | 7e-19 |
| AGO86666.1 | putative\_MFS\_transporter | BGC0001255 | Equisetin\_biosynthetic\_gene\_cluster | 39 | 23.1464737794 | 87 | 5e-17 |
| CCT67996.1 | bikaverin\_cluster-efflux\_pump | BGC0000030 | Bikaverin\_biosynthetic\_gene\_cluster | 34 | 24.2314647378 | 82 | 2.1e-15 |
| CCH26293.1 | putative\_MFS-type\_transporter | BGC0001158\_c4 | Bikaverin\_biosynthetic\_gene\_cluster | 33 | 24.2314647378 | 81 | 4.7e-15 |
